# A longitudinal study of the prevalence and characteristics of breast disorders detected by clinical breast examination during pregnancy and six months postpartum in Ibadan, Southwestern Nigeria

**DOI:** 10.1186/s12905-018-0647-4

**Published:** 2018-09-19

**Authors:** Stella O. Odedina, IkeOluwapo O. Ajayi, Adenike Adeniji-Sofoluwe, Imran O. Morhason-Bello, Dezheng Huo, Olufunmilayo I. Olopade, Oladosu A. Ojengbede

**Affiliations:** 1 Department of Epidemiology and Medical Statistics, Faculty of Public Health, University of Ibadan, Ibadan, Nigeria; 2 Department of Radiology, Faculty of Clinical Sciences, University of Ibadan, Ibadan, Nigeria; 3 Centre for Population and Reproductive Health, College of Medicine, Ibadan, Nigeria; 4 Department of Obstetrics and Gynaecology, Faculty of Clinical Sciences, College of Medicine, University of Ibadan, Ibadan, Nigeria; 5 Department of Public Health Sciences, University of Chicago, Chicago, IL USA; 6 Center for Global Health, University of Chicago, Chicago, IL USA

**Keywords:** Benign breast diseases, Pregnant women, Antenatal clinics, Longitudinal, Clinical breast examination, Post-partum

## Abstract

**Background:**

Breast disorders cause great anxiety for women especially when they occur in pregnancy because breast cancer is the most common cause of cancer related deaths in women. Majority of the disorders are Benign Breast Diseases (BBD) with various degrees of associated breast cancer risks. With increasing breast cancer awareness in Nigeria, we sought to determine the prevalence and characteristics of breast disorders among a cohort of pregnant women.

**Methods:**

A longitudinal study of 1248 pregnant women recruited in their first trimester- till 26 weeks gestational age consecutively from selected antenatal clinics (ANCs), in Ibadan, Southwest Nigeria. A pretested interviewer- administered questionnaire was used to collect information at recruitment. Clinical Breast Examination (CBE) using MammaCare**®** technique was performed at recruitment and follow up visits at third trimester, six weeks postpartum and six months postpartum. Women with breast disorders were referred for Breast Ultrasound Scan (BUS) and those with Breast Imaging Reporting and Data System (BIRADS) ≥4 had ultrasound guided biopsy. Statistical analysis was performed using Stata version 14.

**Results:**

Mean age of participants was 29.7 ± 5.2 years and mean gestational age at recruitment was 20.4 ± 4.4 weeks. Seventy-two participants (5.8%) had a past history of BBD and 345 (27.6%) were primigravidae. Overall, breast disorder was detected among 223 (17.9%) participants and 149 (11.9%) had it detected at baseline. Findings from the CBE showed that 208 (69.6%) of 299 breast disorders signs found were palpable lumps or thickenings in the breast, 28 (9.4%) were persistent pain, and 63 (21.1%) were abscesses, infection and mastitis. Twenty out of 127 (15.7%) participants who had BUS performed were classified as BIRADS ≥3. Lesions found by BUS were reactive lymph nodes (42.5%), prominent ducts (27.1%), fibroadenoma (9.6%), breast cysts (3.8%) and fibrocystic changes (2.5%). No malignant pathology was found on ultrasound guided biopsy.

**Conclusions:**

Breast lump is a major breast disorder among pregnant women attending antenatal clinics in Ibadan. Routine clinical breast examination and follow up of pregnant women found with breast disorders could facilitate early detection of pregnancy associated breast cancer in low resource settings.

## Background

Breast disorders are very common among women [1–3] and some are known to be associated with increased risk for breast cancer [4–6]. Few studies have looked into benign breast diseases in sub-Saharan Africa [4]. In Nigeria, most studies have focused on histopathology characteristics of benign breast disease [7, 8]. Few studies have explored the clinical diagnosis of breast disorders [3, 9].

During pregnancy, many women suffer from different types of breast problems including fibroadenoma, mastitis, breast abscess, galactocoele and pregnancy associated breast cancer (PABC) which is very common because of the relative young age at breast cancer diagnosis in the population [9–13]. Pregnant women with breast problems should be investigated thoroughly and this should be done at regular intervals [10, 12, 14]. Pregnancy associated breast cancer even though rare is known to be very aggressive with poor outcomes attributed to diagnostic delays [12, 15, 16].

Breast cancer screening is a viable option for early detection of breast cancer [3]. In sub-Saharan Africa, screening programmes are non-organized and implementation of such programmes has been very challenging [9, 17, 18]. Breast screening approach requires triple assessment before excluding breast cancer, and this is also applicable in pregnancy. Clinical breast examination (CBE) should not be done alone, further investigations such as Breast Ultrasound Scan and ultrasound guided biopsy are required and are safe for pregnant and lactating women [9, 14, 19, 20].

Our study is the first to examine women for breast disorders at intervals in pregnancy and postpartum using CBE, BUS and ultrasound guided biopsy when necessary and it determined the prevalence and characteristics of breast disorders in pregnancy and 6 months postpartum A similar study was conducted previously in the eastern part of Nigeria and its objective was to compare the detection rate of CBE with BUS among pregnant women [9]. Due to the link or relation between benign breast diseases and breast cancer risk, understanding the pattern of breast disease is an important step towards formulation of policies for breast screening and planning in Nigeria.

The study aimed to determine the prevalence and characteristic of breast disorders as detected by CBE and classified by ultrasonography and ultrasound guided biopsy in pregnancy and six months postpartum. The secondary aim of the study was to identify differences in the characteristics of women with breast disorders and women without breast disorders. The hypothesis was that breast disorders detection in pregnancy or during postpartum can aid early diagnosis of pregnancy-associated breast cancer.

## Methods

### Study setting

Study centers and settings include the antenatal and postnatal clinics of the University College Hospital (UCH), Adeoyo Maternity Hospital (AMH) and Primary Health Centre (PHC) Agbongbon in Ibadan representing tertiary, secondary and primary public health facilities based on three level of care in Ibadan, Southwestern Nigeria. Ibadan is the capital and a city in Oyo State, Nigeria. Oyo state has a population of 5,580,894 according to 2006 census and 1,633,333 are reproductive age women (15-49 years) [21]. Therefore, Ibadan metropolis with a population of 1, 343, 147 has an estimate of 392,733 reproductive age women. Majority of the people in Ibadan are of Yoruba ethnicity. Both UCH and AMTH are main public health care facilities rendering maternal care services to majority of women in the city.

### Study design and study population

This is a longitudinal study of pregnant women attending antenatal clinics (ANCs) in Ibadan, Nigeria. All pregnant women within first trimester till- 26 weeks gestational age were eligible. Pregnant women with previous breast cancer history were excluded.

### Sample size and sampling

The study sample size of 970 was calculated using the sample size formula for estimating relative risk [22]. Several proportions of relative risk factors for breast diseases were calculated and the highest sample size using age at menarche with a relative risk of 0.80 as estimate was picked [23]. The sample size was calculated assuming a type 1 error of 5% and accounting for a non-response rate of 20%.

After obtaining informed consent, participants were consecutively recruited during their ≤26 weeks gestational age. They were followed up in their third trimester, six weeks postpartum and six months postpartum considering variations in hormones and the difficulty in detecting lumps in pregnancy and during lactation.

### Data collection methods

A pretested and face validated questionnaire was used to collect information on socio-demographic characteristics, medical history, lifestyle and reproductive variables. Test-retest method was used to ascertain the reliability of the questionnaire. The questionnaire was administered to ten pregnant women twice with two-week interval at a different health centre (PHC Bashorun) from the study centre. Variables from the questionnaires were computed. Total composite scores for pre-test and post-test were then compared. A correlation value of 0.9 was achieved.

Research nurses and research assistants were trained prior to the commencement of the study. Research Nurses were introduced to the study and instructed on breast health and MammaCare**®** method of Breast Self Examination (BSE) and CBE. MammaCare**®** method is validated and recommended for effective CBE and BSE [24]. It involves the use of tactile senses and it follows 5P rule for complete breast examination. The 5Ps are palpation, pressure, pattern, perimeter and position. The pads of three middle fingers are used for palpation, three levels of pressure are applied at a spot (light, middle, and deep), breasts are examined using a vertical strip pattern movement, perimeter of the breast, extending to the axilla is outlined and examined, and finally, both breasts should be examined in the cahan and supine positions.

Trained Research nurses practiced on breast models and examined human volunteers attending ANC at the University College Hospital, Ibadan. During the training, the Clinical Breast Examination tool, which included a checklist, was developed and reviewed to ensure consistency and reduce selection bias while defining breast disorders. In addition, inter rater reliability analysis was performed by rating each nurse performance and agreement on detecting lumps on a breast model. The inter-rater reliability for the raters kappa coefficient was 0.55 (*p* < 0.001), which represents a moderate agreement among the Nurses.

The study spans from June 2015 to September 2017. All study participants were taught how to examine their breasts. Clinical breast exam using MammaCare**®** technique was performed by trained nurses at recruitment and at follow up visits in all centers. Research assistants administered the questionnaire after obtaining informed consent at recruitment. Participants were considered to have breast disorders if breast changes such as palpable lumps, inflammation, persistent pain and/or abnormal nipple discharge were found using the checklist. Those with breast disorders were referred for BUS and ultrasound guided biopsy as necessary. All study participants were taught how to examine their breast at recruitment.

Breast ultrasound scan was performed by a consultant radiologist at the Radiology Department of UCH, and a private facility located in Ibadan. The BUS assessment was made using Breast Imaging Reporting and Data System (BIRADS). Ultrasound guided biopsy was performed for those with BIRADS 4 lesion by a Consultant Radiologist and biopsy read at the Pathology Department of UCH.

### Statistical analysis

Descriptive statistics was used for calculating the percentages. Generalized Linear Marginal Model (GLMM) with Generalized Estimating Equation (GEE) was used to determine the trend of breast disorders in pregnancy and in lactation and it was also used to determine association between selected patients characteristics with breast disorder status. The GLMM assumed an exchangeable variance covariance structure. Variables whose *p*-value was < 0.05 from bivariate analysis were selected into the final GLMM.

All statistical analyses were done using Stata version 14 (Texas, USA).

Ethical approval was gotten from the University of Ibadan/University College Hospital (UI/UCH) Ethics Committee and the Oyo State Ministry of Health Ethics Committee, Ibadan, Nigeria and all participating women gave their written informed consent.

## Results

### Characteristics of study participants

A total of 1248 pregnant women within ages 16–46 were recruited. The mean ± SD age was 29.7 ± 5.2 years and mean ± SD gestational age at recruitment was 20.4 ± 4.4 weeks. Seven hundred and eight (56.6%) women had above secondary level education. All study participants were of African heritage with majority (92.5%) from Yoruba ethnic group, 72 (5.8%) had a history of breast disease, 345 (27.6%) were primigravidae and 58 (4.7%) had family history of cancer (Table 1).

**Table 1:** Characteristics of study participants

	Breast Disorders	Breast Disorders	Total
	(yes) *N* = 223	(no) *N* = 1025	*N* = 1248
	*n* (%)	*n* (%)	
Age group (years)			
< 25	55 (24.7)	149 (14.5)	204 (16.4)
25–29	75 (33.6)	337 (32.9)	412 (33.0)
30–34	58 (26.0)	325 (31.7)	383 (30.7)
> 34	35 (15.7)	214 (20.9)	249 (19.9)
Health Facility			
PHC	25 (11.2)	77 (7.5)	102 (8.2)
AMH	160 (71.8)	625 (60.9)	785 (62.9)
UCH	38 (17.0)	323 (31.5)	361 (28.9)
Education			
None/Primary	22 (9.9)	62 (6.1)	84 (6.7)
Secondary	105 (47.1)	351 (34.2)	456 (36.5)
Polytechnic	47 (21.1)	215 (20.9)	262 (20.9)
B. Sc./HND	42 (18.8)	303 (29.7)	345 (27.6)
Postgraduate	7 (3.1)	94 (9.2)	101 (8.1)
Ethnicity			
Yoruba	206 (92.4)	948 (92.5)	1154 (92.5)
Ibo	9 (4.0)	39 (3.8)	48 (3.9)
Hausa	3 (1.4)	9 (0.9)	12 (0.9)
Others	5 (2.2)	29 (2.8)	34 (2.7)
Occupation^*^ *N* = 1247			
Professionals	8 (3.6)	88 (8.6)	96 (7.7)
Civil servants	47 (21.1)	342 (33.4)	389 (31.2)
Full time housewife, unemployed	26 (11.7)	99 (9.7)	125 (10.0)
Artisan, small scale entrepreneur,			
labourers	142 (63.7)	495 (48.3)	637 (51.1)
Family history of any cancer type			
Yes	11 (4.9)	47 (4.6)	58 (4.6)
No	212 (95.1)	978 (95.4)	1190 (95.4)
Ever had benign breast disease			
Yes	19 (5.2)	53 (5.2)	72 (5.8)
No	204 (91.5)	972 (94.8)	1176 (94.2)
No of pregnancies			
1	72 (32.3)	273 (26.6)	345 (27.6)
2	59 (26.5)	249 (24.3)	308 (24.7)
> 2	92 (41.3)	503 (49.1)	595 (47.7)
Age at menarche (years)^*^ *N* = 1239	15.3 ± 2.0	15.1 ± 2.3	15.1 ± 2.2
Height^*^ *N* = 1211	158.5 ± 6.7	159.4 ± 6.6	159.3 ± 6.6
Weight (kg)^*^ *N* = 1209	63.9 ± 12.2	67.5 ± 13.0	66.8 ± 12.9
BMI kg/m^2*^			
< 18	6 (2.8)	12 (1.2)	18 (1.5)
18–24.9	109 (50.7)	423 (42.9)	532 (44.3)
25–29.9	60 (27.9)	318 (32.2)	378 (31.5)
30–34.9	32 (14.9)	176 (17.8)	208 (17.3)
> 34.9	8 (3.7)	58 (5.9)	66 (5.5)

*PHC* Primary Health Centre Agbongbon, *AMTH* Adeoyo Maternity Teaching Hospital, *UCH* University College Hospital, ^*^missing N ≠ 1248

One hundred and eighty three (14.7%) of study participants had ever done CBE prior to the study, but only 50 (27.3%) from these women had it done during ANC booking or registration. Other places mentioned were: whenever they noticed any breast change and they made breast complaint to health practitioners 55 (30.1%), during normal routine check up 20 (10.9%), and during breast health sensitization or seminar programmes in their community, place of worship or place of work 8 (4.4%).

### Prevalence and pattern of breast disorders CBE findings

**Fig. 1:**
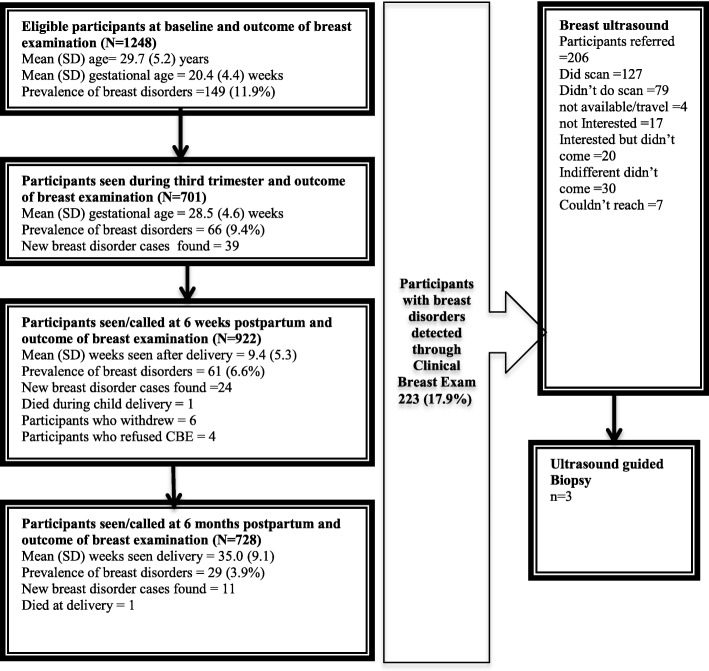
Participants flow chart with proportion seen at various visits

**Fig. 2:**
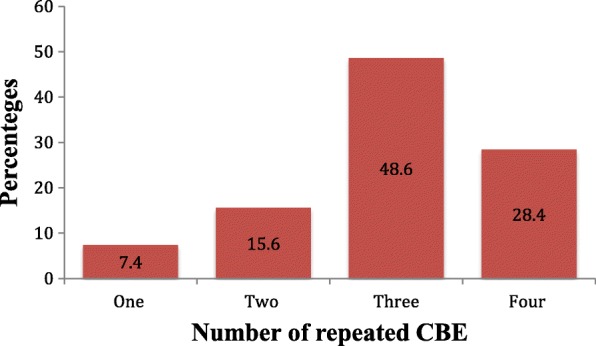
Repeated CBE as experienced by study participants *N* = 1248

**Fig. 3:**
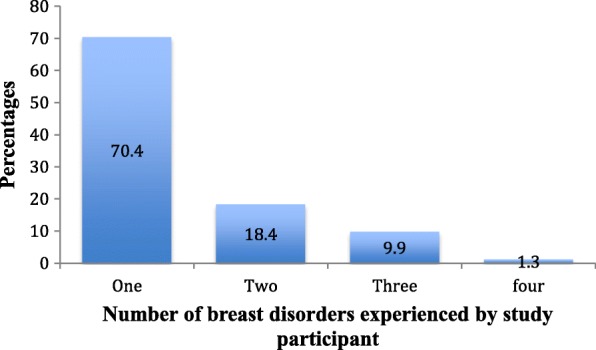
Breast disorders as experienced by study participants *N* = 223

All the pregnant women had CBE done during their clinic visit at recruitment and 701 (56.2%) had CBE done in their third trimester. At postpartum, 922 (73.9%) were seen at their various postnatal clinics and those who could not make their clinic visit were called at 6 weeks postpartum also, 728 (58.3%) were seen or called at 6 months postpartum (Fig. 1). Figure 2 shows the frequencies of CBE experienced by study participants, 606 (48.6%) had three CBE and 354 (28.4%) had four CBE. A total of 223 (17.9%) women had breast disorders detected throughout the length of the entire study. At baseline, 149 (11.9%) had breast disorder detected while 74 (6.7%) of those without breast disorders had it detected at follow up. An incidence rate of 9.2 per 100 person years was found. Women found with breast disorders at baseline had a mean age of 28.2 ± 5.4 years. For all women with breast disorders at baseline and follow up, the mean age was 28.5 ± 5.3 years. Palpable masses comprises 208 (69.6%) of all breast disorders detected and 63 (21.1%) were abscesses, infection and mastitis. Table 2 shows the proportion of different breast disorders found at recruitment and at follow up. One hundred and fifty seven (70.4%) women of those found with breast disorders overall in the study had only a form of breast disorders while 66 (29.6%) had two or more forms (Fig. 3). Breast disorders were identified on the left breast 87 (39.0%), on the right breast 83 (37.0%), and in both breasts 54 (24.0%).

**Table 2:** Findings from Clinical Breast Examination (CBE) of participants

	Overall *N* = 299	Baseline only *N* = 195
	*n* (%)	*n* (%)
Palpable masses	208 (69.6)	141 (72.3)
Abscesses, Infection, Mastitis	63 (21.1)	38 (19.5)
Persistent pain	28 (9.4)	16 (8.2)
Palpable masses with pain ^a^	15 (5.0)	6 (3.1)
Infection with pain ^a^	4 (1.3)	2 (1.0)

^a^Overlapping signs and symptoms

### Breast ultrasound scan findings

One hundred and twenty seven (61.7%) women referred did BUS. Eighteen (14.2%) had BIRADS 3 and 98 (77.2%) had BIRADS 2 (Table 3).

**Table 3:** Ultrasound Breast Imaging Reporting and Data System (BIRADS) classification of breast disorders (*N* = 127)

BIRADS Categories	Descriptors	*n* (%)
BIRADS 0	Needs additional imaging evaluation	0 (0.0)
BIRADS 1	Negative	9 (7.1)
BIRADS 2	Benign findings	98 (77.2)
BIRADS 3	Probably benign findings; short interval follow up suggested	18 (14.2)
BIRADS 4	Suspicious abnormality; biopsy should be considered	2 (1.6)
BIRADS 5	Highly suggestive of malignancy; appropriate action should be taken	0 (0.0)
BIRADS 6	Known biopsy-proven malignancy; appropriate action should be taken	0 (0.0)

**Fig. 4:**
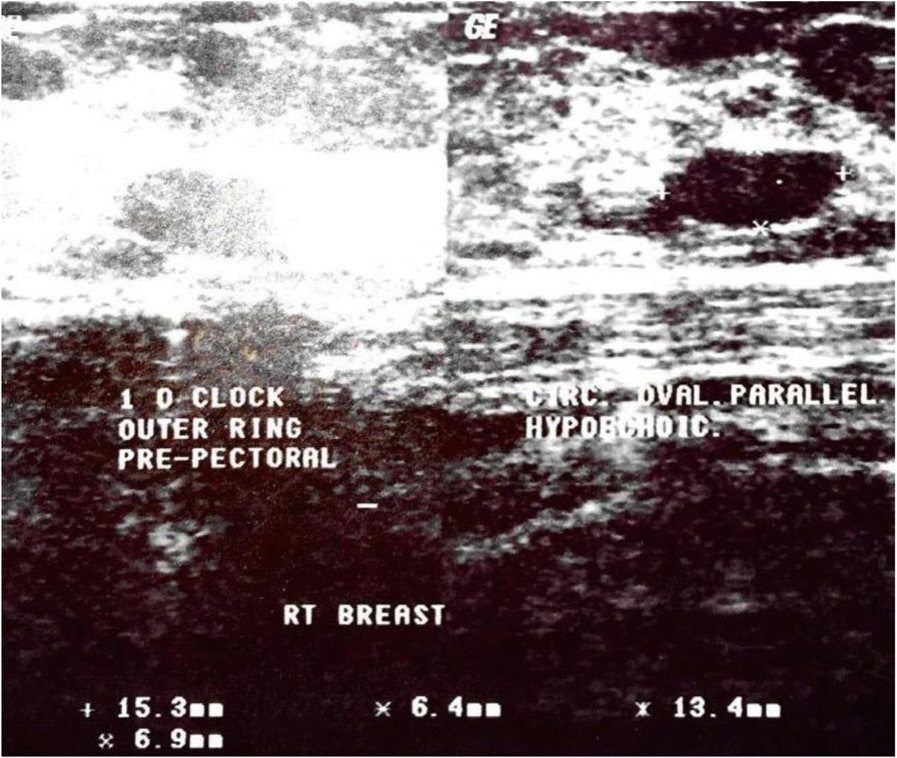
Circumscribed oval hypoechoic masses in parallel orientation in the right breast seen at 1 O′ clock position in the middle-outer ring and pre-pectoral region and at 12 O′ clock position in the inner ring with posterior enhancement- fibroadenoma and breast cysts

Three women whose BUS reports were suspicious of malignancy did ultrasound-guided biopsy. Impressions from ultrasound findings showed that most had reactive lymph nodes (42.5%) and prominent ducts (27.1%). Other findings were fibroadenoma (9.6%), fibrocystic changes (2.5%) and breast cysts (3.8%). Figure 4 shows a BUS image of a woman diagnosed with fibroadenoma and breast cyst. Forty four (34.6%) masses or structures were found by the scan from the 127 that had BUS done, of which 14 (31.8%) were found to be on the left breast, 13 (29.5%) on the right breast and 17 (38.6%) in both breasts. Table 4 shows in detail, impressions from the BUS based on time detected in pregnancy or lactation by CBE. The median time from CBE at recruitment to BUS was 191 ± 141 days. The period between breast disorder detection by CBE and having BUS done was long as depicted by the median time above. This must have accounted for a lesser proportion of masses found by BUS compared to CBE.

**Table 4:** Findings from Breast ultrasound scan reports showing proportion of breast disorders detected by CBE at recruitment and follow up

BUS Impressions	Recruitment	Third trimester	Six weeks postpartum	Six months postpartum
	*n* (%)	*n* (%)	*n* (%)	*n* (%)
Reactive lymph nodes	70 (44.0)	10 (50.0)	10 (47.6)	2 (22.2)
Prominent ducts	39 (24.5)	7 (35.0)	6 (28.6)	4 (44.4)
Fibro adenoma	19 (11.9)	2 (10.0)	1 (4.8)	0 (0.0)
Fibrocystic changes	4 (2.5)	1 (5.0)	0 (0.0)	0 (0.0)
Breast cyst/complex cyst	5 (3.1)	0 (0.0)	2 (9.5)	0 (0.0)
Infected dermal inclusion cyst/ sebaceous cyst	4 (2.5)	0 (0.0)	1 (4.8)	1 (11.1)
Duct ectasia	0 (0.0)	0 (0.0)	0 (0.0)	1 (11.1)
Galactocoele	3 (1.9)	0 (0.0)	0 (0.0)	1 (11.1)
Mastitis	1 (0.6)	0 (0.0)	0 (0.0)	0 (0.0)

### Ultrasound guided biopsy

No malignant pathologies were found in the biopsy specimen of the three women referred for breast ultrasound guided biopsy. Diagnoses made were florid adenosis, lactational adenoma and fibroadenoma/pregnancy associated changes with infection.

### Changes in the breast with time and associated patients characteristics

The prevalence of breast disorders detected by CBE reduces with time on the generalized linear model with GEE (*p* < 0.001). Compared to the risk of detecting breast disorders by CBE at recruitment, the odds ratio for breast disorders was 0.80 (95% CI: 0.62–1.03) at third trimester, 0.54 (95% CI: 0.41–0.69) at 6 weeks postpartum and 0.35 (95% CI:0.25–0.49) at 6 months postpartum.

Table 5 shows the association between selected patients characteristic and breast disorders in pregnancy and six months postpartum adjusting for variables significant at 5% (*p* = 0.05) from bivariate analysis and some important variables. Variables included in the final GLM model were time, age group, number of pregnancies, study centre, weight, education, occupation, ever had history of benign breast diseases, BMI (group) and relative history of any cancer type. Women who had B.Sc. equivalent level of education had lower odds of breast disorders compared to those with none or primary level of education (aOR = 0.47, 95% CI = 0.24–0.89), women with a history of benign breast diseases had higher odds of breast disorders compared to those without the history (aOR = 2.13, 95% CI = 1.25–3.64), and women with three or more pregnancies had higher odds of breast disorders compared to those with one. (aOR = 0.65, 95% CI = 0.43–0.97).

**Table 5:** Association of selected patients characteristic with breast disorders in pregnancy and six months postpartum

	Adjusted Odds ratio	95% Confidence Interval	*p*-Values
Age group (years)			
< 25	1(ref)		
25–29	1.07	0.71–1.63	0.737
30–34	0.98	0.60–1.59	0.941
> 34	0.89	0.52–1.54	0.682
Health Facility			
UCH	1(ref)		
AMH	1.03	0.63–1.67	0.918
PHC	0.79	0.38–1.65	0.535
Education			
None/Primary	1(ref)		
Secondary	0.75	0.45–1.26	0.281
Polytechnic	0.63	0.35–1.13	0.120
B. Sc./HND	0.47	0.24–0.89	0.022**
Postgraduate	0.41	0.14-1.18	0.100
Occupation			
Professionals	1(ref)		
Civil servants	1.69	0.67–3.77	0.252
Full time housewife, unemployed	2.70	1.05–6.95	0.039**
Artisan, small scale entrepreneur,			
labourers	2.00	0.79–5.08	0.143
Family history of cancer			
No	1(ref)		
Yes	1.53	0.76–3.06	0.230
Ever had benign breast disease			
No	1(ref)		
Yes	2.13	1.25–3.64	0.006**
No of pregnancies			
1	1(ref)		
2	0.91	0.61–1.37	0.665
> 2	0.64	0.43–0.97	0.036**
Weight (kg)	0.99	0.96–0.99	0.085
BMI kg/m^2^			
18–24.9	1(ref)		
< 18	1.56	0.49–4.95	0.451
25–29.9	0.93	0.59–1.47	0.766
30–34.9	1.30	0.66–2.58	0.448
> 34.9	1.28	0.37–4.46	0.695

**Significant at *P* < 0.05

## Discussion

Benign breast diseases are very common among young women in their reproductive ages, irrespective of whether pregnant, lactating or not [1, 2]. The mean age of women with breast disorders in this study was 28.5 years. This falls within the age range of women with benign breast diseases in other studies conducted respectively in Warri, Benin, Lagos and Ile-Ife, Nigeria [8, 25–27].

At baseline, the prevalence of breast disorders in pregnancy detected by CBE was 11.9%. Throughout pregnancy and 6 months postpartum, the total prevalence of breast disorder was 17.9%. Findings from some community surveys in Africa, whereby breast screening was carried out among women in general reported lesser proportions. For example, a study conducted in Ghana found 4.8% of 1419 premenopausal women had clinically palpable breast lumps of which screening was conducted by mammocare**®** experts [28]. Abuidris, et al. [17] in a house to house breast screening pilot survey in Sudan reported 1.3% of their participants to have breast abnormalities detected by trained local volunteers. However, a prevalence of 5.3% of breast lesions detected by repeat CBE in pregnancy and postpartum was reported by an analytical cross-sectional study conducted among pregnant women during antenatal clinic in Abakaliki teaching hospital, Ebonyi, Nigeria [9], a higher proportion of women with breast disorders detected through CBE was found in this study.

A higher prevalence of breast disorders in pregnancy than non-pregnant state is not unusual because of the influence of female’s hormones and growth factors causing several physiological changes during pregnancy and lactation [12, 29, 30]. Reasons for a higher prevalence in our study could be due to longitudinal design that gave more time for more breast disorders to be detected. Another plausible reason is the inter-observer variation by nurses on use of MammaCare**®** for CBE at the 3 study centers. Despite these possibilities, Mammacare**®** techniques used has been scientifically validated for CBE and it has been proven to detect lumps as small as 0.3 cm [24].

In Nigeria, majority of breast cancer cases are known to occur among women in their reproductive ages and the cancer types associated has been proven to be very aggressive [1, 2, 31]. A rise in the incidence of breast disease in Nigeria has also been reported [25] and the incidence is likely to increase due to the increase in breast cancer awareness. About 20% of pre-menopausal breast cancer cases occurred during pregnancy and within 5 years postpartum in a Nigerian study [32]. An incidence rate of 9.2 per 100 person years for breast disorders was noted in our study. Therefore, close attention and monitoring of pregnant and lactating women with breast problems is necessary.

Although the prevalence of women with breast disorders was high, interestingly, the risk of having breast disorders detected by CBE in pregnancy for an individual reduces with time over the period of follow up (third trimester, six weeks postpartum, six months postpartum). Some benign breast diseases may have been masked by the increase in breast density or they underwent secretory, cystic and necrotic changes or they simply regress spontaneously after childbirth. Examples of BBD known for these attributes are fibroadenoma and fibrocystic changes found in some non African studies [12, 33, 34].

A palpable mass is the most common sign of breast disease detected by CBE as reported by studies conducted in South Africa and Rwanda, respectively [35, 36]. This is in line with findings in this study, as palpable mass accounts for 69.6% of all breast disorders. Similarly, the same was reported for breast diseases in pregnancy by a study conducted in eastern Nigeria [9]. Contrary to the above findings, inflammation of the breast was reported to be the most common among women in a Ghana study [28].

A slight left laterality of breast disorders was found in this study. Studies conducted in Ile-Ife, Ilesha, Port-Harcourt in Nigeria, reported a right sided preponderance of benign breast diseases. Their studies however did not indicate whether it was found in pregnancy or not but it was found among women in general [8, 27, 37]. No statistically significant difference was observed between the left or the right laterality of breast cancer and clinical outcome [38]. However, reasons given for the conflicting reports of the laterality of breast cancer were detection bias, handiness of the patients and size differences of the patients breasts [39, 40].

Findings from breast ultrasound scan showed majority of women had reactive axillary lymph nodes and prominent ducts. The prominent ducts are due to physiological changes in the breast during pregnancy and lactation. It appears as hypoechoic tubular structures on b-mode ultrasound scan; it represents ducts filled with colostrum and echogenic milk in late pregnancy and during lactation [13].

Lumps in pregnancy might not necessary be different from non pregnant state [30]. In this study, aside the reactive lymph nodes and prominent duct detected, fibroadenoma was the most frequent breast mass found at recruitment till six weeks postpartum, others were fibrocystic changes, breast cyst and galactocoele. Breast cyst, galactocoele were detected more at six months postpartum. In non pregnant state, several studies have widely reported fibroadenoma to be the commonest in young women [2, 3, 5, 8, 25, 27, 34, 37, 41, 42]. While in some studies conducted in Lagos and Maiduguri, both in Nigeria, it was documented to be second [1, 26] .

Ezeonu et al. [9] also revealed fibroadenoma as the commonest breast disease during pregnancy and lactation in a study conducted in Abakaliki teaching hospital.

According to Bell et al. [11], fibroadenoma was the most common benign solid lesion that grows during pregnancy and breastfeeding due to increase hormones, however, galactocoele was found to be the commonest during lactation after cessation of breastfeeding. Another study reports fibroadenoma to be a common BBD among lactating women which can also present in third trimester, after delivery and breast feeding cessation while lactating adenoma was reported to be the commonest in pregnancy [34]. Joshi et al. [34] described lactating adenoma as masses that can mimic fibroadenoma and phylloides tumour, among others. These masses can reduce in size in third trimester and regress spontaneously after delivery [34]. While some believe lactational adenoma is a unique entity [10], Adeniji-Sofoluwe et al. [13] described sonographic findings of fibroadenoma and lactational adenoma to be indistinguishable but lactational adenoma may show posterior acoustic enhancement increased compressibility.

Even though three participants were referred for biopsy, breast cancer diagnosis was not made in this study. Ezeonu et al. [9] attributed the reason they found no malignant case in their study to participants characteristics such as their young age, young age at first childbirth and high parity. These reasons might be unlikely, as majority of palpable masses in pregnancy are known to be benign with a percentage of 73–88% also breast cancer is found more among young women in Nigeria [12, 43, 44]. Nevertheless masses found during pregnancy should not be delayed for evaluation as there might be increase in PABC due to the trend of delay in child bearing age [34].

An association was found between number of pregnancies and breast disorders in this study. After adjusting for confounders, women with 3 or more pregnancies had lower odds for breast disorders compared to those who had one. This is interesting as increasing parity is an established factor for reduced breast cancer risk among women in general, this was also noted in a Nigerian study [45]. Fibroadenoma risk also reduces with increasing number of full term pregnancy in a study conducted in Ogun state, Nigeria [3].

Other patients’ characteristic associated with lower odds of breast disorders in this study is having a bachelor’s level of education compared to those with none or primary level of education. Variables associated with higher odds for breast disorders were a history of previous benign breast diseases and being a full time housewife or unemployed.

### Limitations of the study

Approximately 40% of women referred for ultrasound scan did not show up, in addition, there were delay from CBE to BUS uptakes. These may affect some of the findings in this study as lower proportion of masses were found in the ultrasound reports compared to CBE. Fear and activities of alternative or traditional medicine are reasons that could account for not appearing for BUS [3]. Misclassification bias arising from different nurses with different level of experience could have affected the definition of breast disorder during breast examination and this might have affected findings in this study. Misclassification bias was however reduced because the nurses were trained together prior to the commencement of this study and the inter-rater agreement was moderate. Also, some participants who were not seen at their 6 weeks postpartum and 6 months postpartum clinic visit were called and asked for any noticeable breast changes to reduce loss to follow up.

## Conclusion and recommendations

This study found a substantial number of pregnant women with breast lumps detected by CBE. We found fibroadenoma to be the commonest breast disease in pregnancy and at six weeks postpartum, while breast cyst and galactocoele were found at six weeks and six months postpartum. The incidence of breast disorders detected by CBE was 9.2 per 100 person years in pregnancy and lactation. The risk of breast disorders detected through CBE reduced with time in pregnancy and lactation.

We recommend breast screening like CBE to be done twice during pregnancy, at ANC registration and once in third trimester. For lactating women, CBE should be done at 6 weeks postpartum. However, those found with breast disorders should be referred immediately for BUS and ultrasound guided biopsy.

Antenatal and postnatal clinics should be considered a place for intensified screening and teaching of breast education. This is a feasible and very useful option for early breast cancer diagnosis. As close to 60% of women in their reproductive ages who attend ANC at least once in pregnancy according to National Demographic Health Survey 2013 [46] would be captured. Due to the challenging nature of examining the breast during pregnancy and lactation, health care workers should be trained on how to conduct thorough CBE using standard and validated techniques. Further diagnostic investigations such as BUS and ultrasound guided biopsy should be conducted after breast disorders are found during CBE. Factors affecting low diagnostic uptake should be addressed by educating the women. Studies with longer follow up duration could have possibly found cases of PABC, in addition, it could as well determine the risk of breast cancer after breast disorder diagnosis.

## Abbreviations

ANC: Antenatal clinics
BBD: Benign breast diseases
BIRADS: Breast imaging reporting and data system
BSE: Breast self examination
BUS: Breast ultrasound scan
CBE: Clinical breast examination
GEE: Generalized estimating equation
GLMM: Generalized linear marginal model
PABC: Pregnancy associated breast cancer
UI/UCH: University of Ibadan/University College Hospital
